# Effects of different preceding crops on soil nutrients and foxtail millet productivity and quality

**DOI:** 10.3389/fpls.2024.1477756

**Published:** 2024-11-25

**Authors:** Chongyan Shi, Tian Qiu, Yangyang Zhang, Yuchao Ma, Xiaorui Li, Shuqi Dong, Xiangyang Yuan, Xi’e Song

**Affiliations:** Key Laboratory of Crop Chemical Regulation and Chemical Weed Control, College of Agronomy, Shanxi Agricultural University, Jinzhong, China

**Keywords:** crop rotation, preceding crop, soil nutrient, agricultural productivity, millet quality, foxtail millet

## Abstract

Crop rotation can affect crop productivity and soil characteristics; however, the impact of preceding crops on the yield and quality of foxtail millet and the relationship between these two factors have not been well characterised. To further investigate the effects of preceding crops on foxtail millet, this study cultivated maize, mung beans, soybeans, potatoes, and proso millet as the preceding crops and rotated them with Zhangzagu10 foxtail millet. A randomised complete block design was employed for the study, and soil and millet samples were collected after harvest. The performance of Zhangzagu10 foxtail millet grown with five different preceding crops was explored by measuring yield and quality indicators and comprehensively analysing various quality traits and their interrelationships. The physicochemical and nutritional characteristics of millet grains were significantly influenced by the preceding crop. The yield of Zhangzagu10 cultivated after mung bean was significantly higher (8277.47 kg/hm*2*) than that of millet cultivated after the other crops. Additionally, the colour characteristics (a*, b*, and △E values) were superior, with the rice exhibiting the strongest yellow colour. Foxtail millet preceded by soybean showed a significantly higher thousand-grain weight, indicating well-filled grains. Furthermore, this treatment had rich contents of carotenoids and polyphenols at 34.79 mg/kg and 76.27 mg/100 g, respectively, and significantly higher levels of minerals such as V, Cr, Fe, Co, Ni, Se, and Sn compared to the other treatments. Foxtail millet following mung bean and soybean demonstrated excellent grain quality, featuring high breakage values and gelatinisation, along with low cooking values and gelatinisation temperatures and moderately low setback values. Zhangzagu10 cultivated after potato exhibited a polyphenol content of 67.13 mg/100 g, showcasing strong antioxidant effects. In contrast, proso millet preceded by foxtail millet had relatively lower content levels across various substances, resulting in an overall subpar performance. In summary, selecting appropriate preceding crops can significantly enhance both the yield and quality of Zhangzagu millet. Moreover, soybeans, potatoes, and mung beans can be effectively incorporated into a sustainable crop rotation plan for millet. In the future, we aim to further explore the interaction mechanisms between preceding crops and millet to optimise rotation strategies and improve foxtail millet quality.

## 1 Introduction

Foxtail millet (*Setaria italica*) is an important coarse- and small-grain cereal crop characterised by cold and drought tolerance that is cultivated worldwide (Balasubramanian et al., 2020). Foxtail millet is rich in starch, vitamins, and various micronutrients (Šenk et al., 2023). With increasing global population growth and economic development, there is an urgent need to enhance both the yield and quality of foxtail millet. Owing to its beneficial effects on human health, the quality of foxtail millet has garnered increasing attention in recent years, particularly in Asia and Africa, where it plays a crucial role in food security (Liang et al., 2018; Johnson et al., 2019). The “Zhangzagu” series of foxtail millet, developed by the Zhangjiakou Academy of Agricultural Sciences in Hebei Province, China, exhibits broad adaptability, high quality, and productivity. These varieties are now cultivated across large acreages in Shanxi, Hebei, and Inner Mongolia in China (Weng et al., 2020; Song et al., 2018).

Continuous cropping refers to the agricultural practice of planting the same crop repeatedly on the same piece of land (Tan et al., 2021). In China, facility cultivation is dominated by soil culture and extremely intensive and long-term single continuous cropping. Continuous monoculture often deteriorates the physical, chemical, and nutrient properties of the soil (Gu et al., 2022). Studies have shown that long-term continuous soybean cropping exacerbates fungal diseases, resulting in a fungal community structure that is unfavourable for plant health (Liu et al., 2019). Continuous cropping also leads to a lower soil pH, available potassium content, and urease activity (Li et al., 2021). It has also been demonstrated that long-term continuous cropping reduces both the yield and quality of cucumbers (Sun et al., 2021). Monoculture, including foxtail millet cultivation, has become the most common intensive agricultural production practice. Consecutive cultivation of foxtail millet results in reduced chlorophyll content in the leaves, affecting photosynthesis and diminishing its capacity for dry matter accumulation. Decreases in foxtail millet yield are even more pronounced with prolonged monoculture (Wen-we, 2013). Proper crop rotation can alleviate the drawbacks of monoculture, with long-term rotation improving soil nitrogen storage, mineralisation, and availability (Fu et al., 2019), which has been shown to promote the growth and yield of young apple trees, as well as enhance fruit quality (Wang et al., 2021). Meanwhile, the rotation of foxtail millet with soybeans and potatoes increases the activities of superoxide dismutase, peroxidase, polyphenol oxidase enzymes, photosynthetic efficiency, millet yield, and disease resistance.

The impact of preceding crops on soil characteristics and crop quality has long been reported. Many previous studies have indicated that the yield and quality of succeeding crops are influenced by the type of the preceding crop (Sainio et al., 2019). Leguminous crops, known for their nitrogen-fixing properties (Zhong et al., 2022), have been widely incorporated into crop rotation systems worldwide (Barbieri et al., 2023). As preceding crops, fava beans can significantly increase the yield and quality of tomatoes, particularly in terms of calcium content (Raffa et al., 2022), legumes can enhance the yield and quality of forage crops (Hassan et al., 2022), and soybean can increase the grain protein content, Zeleny sedimentation value, and grain uniformity (Gawęda and Haliniarz, 2021). Moreover, legume crops can enhance soil fertility, even with minimal nitrogen fertiliser application (Marchetto and Power, 2020). Some grass species can also serve as preceding crops. For example, when wheat is grown as a preceding crop to chrysanthemums, their total flavonoid, chlorogenic acid, and soluble sugar contents increase (Xin et al., 2015), although such effects may be less favourable in some crop rotation systems. When maize precedes wheat, the risk of Fusarium wilt disease is increased (Tillmann et al., 2016). Potatoes, which are members of the family Solanaceae, may also be included in rotation systems. Under long-term conventional management in Switzerland, the effects of preceding potatoes have been superior to those of organic fertilisation, resulting in an increased yield and crude protein content in wheat crops (Mayer et al., 2015).

The quality of foxtail millet, including its appearance, cooking behaviour, and function, are key factors that affect consumer purchasing decisions (Ma et al., 2023). Although extensive research has been conducted on foxtail millet quality, the holistic effects of crop rotation on foxtail millet have received limited attention. The nutritional composition and attributes of foxtail millet are influenced not only by genetic factors but also by cultivation practices. For example, organic farming can enhance the accumulation of fructose and glucose in foxtail millet grains (Liang et al., 2018), and different planting ratios have varying effects on foxtail millet yields and characteristics (Bennett et al., 2011). Yogi et al. (2023) reported that intercropping legumes with foxtail millet cultivation significantly improved millet productivity and protein, oil, Fe, Zn, and Mn contents compared to traditional monoculture. In a maize–soybean–millet rotation system, foxtail millet exhibited significantly higher levels of total amino acids, crude protein contents, and viscosity than under continuous cropping. Complex correlations among different quality traits within millet have been documented (Ma et al., 2023). However, the influence of different preceding crops on the quality of the foxtail millet remains unclear (Smith et al., 2017).

In this study, we employed the Zhangzagu10 foxtail millet variety as the research subject and investigated five different crop rotation systems: (1) maize–foxtail millet, (2) mung bean–foxtail millet, (3) soybean–foxtail millet, (4) potato–foxtail millet, and (5) proso millet–foxtail millet. Our main objectives were to examine the impact of the five preceding crops on the soil nutrient status, foxtail millet yield and quality, and the associations among grain quality traits. The results will provide a theoretical basis for the rational rotation of foxtail millet and offer practical guidance for agricultural production.

## 2 Materials and methods

### 2.1 Study area

Experiments were conducted between May of 2021 and October of 2022. The test site was located on the Haifeng Farmland (39.188°N, 113.606°E) in Jinshanpu Township, Fanshi County, Shanxi Province, China. This region has a temperate continental climate and mean annual temperature and precipitation of 11.6°C and 400 mm, respectively, and the main soil type is sandy loam.

### 2.2 Experimental design

Five preceding crops (maize, mung beans, soybeans, potatoes, and proso millet) were sown in May of 2021, with three replicates each. The plot size was 30 m^2^, and a randomised full-block design was implemented. Typical sowing and field management methods used in the area were followed. Soil samples were collected after harvesting the preceding crops in early October. Samples were then taken to the laboratory for nutrient testing. In May of 2022, the same experimental methods were employed in the planting of foxtail millet, and seed and quality testing were conducted after the foxtail millet was harvested.

### 2.3 Determination of soil nutrient status

The collected soil samples were air-dried at room temperature and screened using a 2-mm sieve to remove large particles and impurities.Using a soil nutrient analyser (IN-CT02, Lai Yin, China) to assesse the organic matter (OM), total nitrogen (TN), total phosphorus (TP), and total potassium (TK) contents for samples across all replicates.

### 2.4 Quality of foxtail millet appearance

The length (L), breadth (B), length–width ratio (L/B), and 1,000-grain-weight (KGW) after shelling were measured using an automatic seed testing instrument (SC-G, Wan Shen Testing Co., China). The brightness (L*), red–green value (a*), and yellow–blue value (b*) of foxtail millet grains after shelling were measured using a colourimeter (WSF, Shanghai Precision Science Instrument Co., Ltd., China). All experiments were repeated in triplicate. The colour differences (ΔE) were then calculated as follows (Zhang et al., 2022):


ΔE=[2(L0−L*)+2(a0−a*)+2(b0−b*)]12

where *L*_0_ = 100, *a*_0_ = 0, and *b*_0_ = 0.

### 2.5 Cooking quality of foxtail millet

#### 2.5.1 Gel consistency

Two samples of approximately 100 mg ± 1 mg foxtail millet flour were placed in test tubes, and then 0.2 mL of 0.025% thymol blue ethanol solution was added. The test tubes were ten gently shaken or placed in a vortex mixer to ensure that the foxtail millet flour was thoroughly dispersed. Next, 2.0 mL of 0.2 mol/L potassium hydroxide solution was added, and the test tubes were shaken to achieve a uniform mixture. Once the foxtail millet flour was fully mixed, the test tubes were immediately placed in a boiling water bath, with the openings covered with glass beads. The tubes were heated in the boiling water bath for 8 min (timing is started as soon as the test tubes are placed in the bath), ensuring that the level of the millet gel solution remains between 1/2 and 2/3 of the height of the test tubes during heating. After 8 min, the test tubes were removed, the glass beads were removed, and the tubes were cooled for 5 min. Then, the test tubes were placed in an ice water bath at approximately 0°C for 20 min. Once cooled, the test tubes were removed from the ice water bath and immediately laid horizontally on a level surface marked with a scale, with the bottoms of the test tubes aligned with the starting line. The test tubes were allowed to sit at 25 ± 5°C for 1 h before measuring the length of the foxtail millet gel flow within the tubes.

#### 2.5.2 Cooking characteristics

We weighed 4 g of foxtail millet (W_0_), washed it with distilled water five times, and then boiled it in a pot containing 200 mL of distilled water at 100°C for 15 min to obtain foxtail millet porridge. A filter was placed in a beaker to separate the foxtail millet from the soup. After filtering, the foxtail millet was allowed to stand for 20 min and then weighed (W_1_). The volumes of foxtail millet (V_0_) and filtered millet porridge (V_1_) were measured via draining. The water absorption rate (WAR, %) and expansion rate (ER, %) were then calculated using the following formulas:


$$WAR=\frac{W_{1}-W_{0}}{W_{0}}\times 100\%$$

$$ER=\frac{V_{1}-V_{0}}{V_{0}}\times 100\%$$

The pH of the soup was measured after it had cooled to room temperature. Using distilled water as a control, the absorbance at λ = 620 nm was measured as the absorbance of the millet soup (LAV) using a UV spectrophotometer (UV 2400, Sunny Heng-ping Instrument, LLC, China). We placed 2 mL of millet soup in a centrifuge tube, centrifuged it at 8000 rpm for 10 min (D-37520, Sigma, Germany), and then collected 0.5 mL of the supernatant, to which we added 15 mL of distilled water. The pH was adjusted to ~3.5 using 1 mol of L-1 HCl; we then added 0.5 mL of 0.2 mol of L-1 iodine reagent and adjusted the volume to 50 mL using distilled water, before letting it stand for 20 min. An ultraviolet (UV) spectrophotometer was used to measure the absorbance at λ = 620 nm using the same concentration of iodine solution as the reference, which was the iodine blue value (IBV) of millet soup.

A total of 5.00 g of foxtail millet was weighed into a 200 mL conical flask, 75 mL of distilled water was added, and the flask was sealed with plastic wrap. The flask was placed on a constant-temperature magnetic stirrer (C22-WT2218, Midea group company limited, China), set to level 3, and steamed for 15 min. The soup and millet grains were then filtered and separated in the conical flask, and the soup was collected into a 50 mL volumetric flask. A small aluminium box was dried to a constant weight (mass = m_1_), 10 mL of the soup was into the box, and the contents were dried in an oven at 120°C. The weight of the aluminium box with the soup (m_2_) was then determined. Each experiment was repeated in triplicate. The solid content of the foxtail millet soup (SS) was given as follows (Ma et al., 2023):


$$SS=\left(m_{2}-m_{1}\right)/5\times 5\times 1000$$

#### 2.5.3 Foxtail millet paste properties

The millet was crushed using a grinder, sieved through an 80-mesh screen, and stored in a refrigerator at -20°C for testing. Next, 25 mL of distilled water was added to 3 g of crushed foxtail millet in an aluminium container. It was then placed in a rapid visco analyser (RVA; TechMaster, Perten Instruments, Hägersten, Sweden) machine, and the experimental conditions were set in ThermoCline for Windows 10.0(Newport Scientific Pty. Ltd., USA). The rotation speed was set at 960 r/min for 0–10 s then reduced to 160 rpm until the end of the test. An initial temperature of 50°C was maintained for 1 min, then increased to 95°C at a rate of 12°C/min, maintained for 25 min, then decreased back to 50°C at a rate of 12°C/min and maintained for 2 min. The entire process lasted 13 min, during which we recorded the peak viscosity (PV), trough viscosity (TV), final viscosity (FV), peak time (PT), pasting temperature (PTM), derivative parameter breakdown viscosity (BD = PV – TV), and setback viscosity (SB = FV – PV) (Zhong et al., 2005).

### 2.6 Nutritional quality of foxtail millet

#### 2.6.1 Moisture content

Three grams of millet flour was placed in a hot air oven set to 100–105°C. After 2–3 h, the sample was removed and weighed again until a constant weight was achieved. The moisture content (MC) was is calculated as the ratio of the weight difference to the initial weight.

#### 2.6.2 Crude fat

Three grams of millet flour, which was obtained by shelling, crushing, and sieving (60 mesh sieve) foxtail millet seeds, was placed into a filter paper bag, dried, cooled, and weighed. Then, the flour was placed into an installed Soxhlet extractor and extracted with petroleum ether for 8 h. The medicine bag was then removed, dried, cooled to room temperature, and weighed to calculate the crude fat content (EE).

#### 2.6.3 Crude protein

The protein content (CP) in foxtail millet was determined using the Kjeldahl nitrogen method. Approximately 0.3 g of the crushed seed sample was weighed and placed in a boiling tube. Then, 5 mL of concentrated sulfuric acid was added, and the mixture was soaked overnight. The sample was heated until it became transparent, and then the nitrogen content was determined using a Kjeldahl nitrogen analyser. The CP content was calculated by multiplying the nitrogen content in the foxtail millet grain by a factor of 6.25.

#### 2.6.4 Carotenoids

Crushed foxtail millet flour (2 g) was placed in a 50 mL centrifuge tube, and the outer wall of the tube was wrapped with aluminium foil to avoid light exposure. We added 20 mL of water-saturated n-butanol by mixing distilled water and n-butanol in a 1:1 ratio and removing the upper layer after standing. After rapid shaking, the mixture was shaken using a shaker for 3 h (ZQZY-78BV, Zhichu instrument, China). The solution was then centrifuged at 8,000 rpm and 4°C for 15 min, and the supernatant was collected into a new light-protected centrifuge tube. Using water-saturated n-butanol as a control, the absorbance (A) was measured as λ = 450 nm using a UV-visible spectrophotometer. Three replicates were performed for each sample and the total carotenoid (TC) content was determined as:


$$TC=\left[\left(\frac{A}{0.250}\right)\times V\right]/m$$

#### 2.6.5 Polyphenols

The phenolic content (TPC) of the sample was determined using the Folin-Ciocalteu method (Ma et al., 2023), employing gallic acid as the standard to prepare a calibration curve. The polyphenol content of the sample is expressed as the mass of gallic acid (mg) per 100 g of dry weight, with the unit being denoted as mg/100 g.

#### 2.6.6 Flavonoids

The flavonoid content (TFC) of the sample was determined using the NaNO_2_-Al(NO_3_)_3_ method (Ma et al., 2023), and rutin was utilised as the standard to prepare a standard calibration curve. The flavonoid content of the sample is expressed as the mass of rutin (mg) contained in 100 g of dry weight, with the unit being denoted as mg/100 g.

#### 2.6.7 Elemental composition

Microwave digestion inductively coupled plasma–mass spectrometry (SQ-ICP-MS) (Thermo Scientific, USA) was used for elemental analysis. We pretreated 2 g of foxtail millet flour with 10 mL of concentrated HNO_3_ and 1 mL of perchloric acid in a high-temperature-resistant glass vessel and soaked it overnight. The next day, the solution was placed in a graphite digester, and the temperature was increased as follows: 0–30 min at 100°C to 30–50 min at 150°C. Digestion was performed until the solution was clear and bright. The digestion vessel was opened at 150°C to drive off the acid to attain a volume of approximately 1–2 mL; finally, a solution of 10 mL was made with distilled water before testing.

### 2.7 Data analysis

Data in the present study were organised using Excel 2010 v. (Microsoft Corp., USA). Statistical analyses and plotting were performed in SPSS 19.0 (SPSS, Inc., USA), Origin 22.0 (Origin Lab Corp., USA), and R (version 4.1.0; http://www.R-project.org). The least significant difference method was employed for multiple comparisons between groups. All experiments were repeated in triplicate, with results presented as the mean ± standard error. Duncan’s test was employed to identify significant differences in foxtail millet quality indicators among the five treatments, with a significance level set at *p* ≤ 0.05.

## 3 Results

### 3.1 Impacts of different preceding crops on soil nutrients and subsequent foxtail millet yield

**Figure 1:**
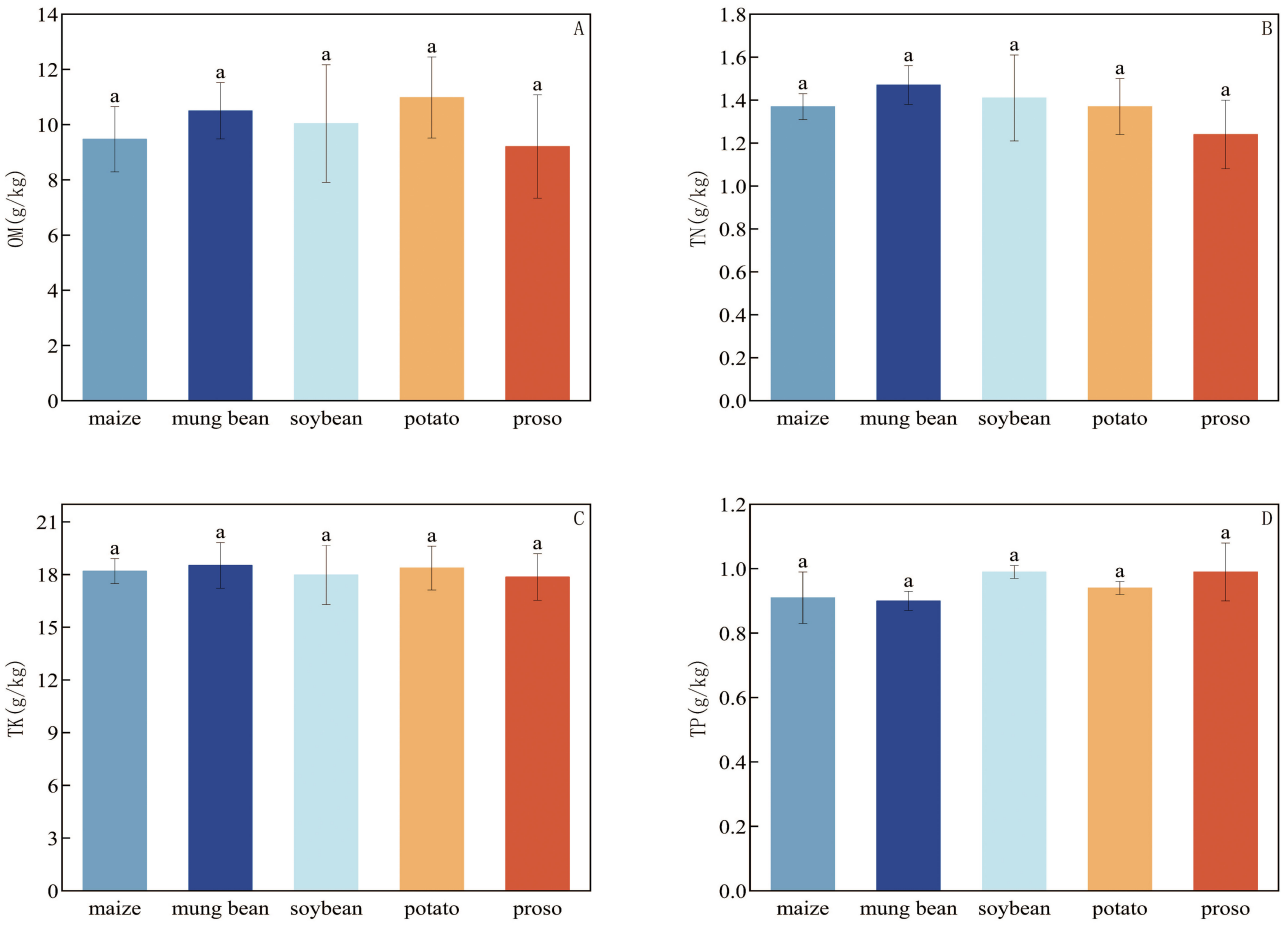
Impacts of different preceding crops on **(A)** organic matter (OM), **(B)** total nitrogen (TN), **(C)** total potassium (TK), and **(D)** total phosphorous (TP). Values are mean ± standard error (SE) of three replicates.Different letters (a, b, c, and d) represented significant differences (p < 0.05).

The nutrient contents of the five soil samples are shown in **Figure 1**, which indicates that no significant differences occurred among the treatments. 

**Figure 2:**
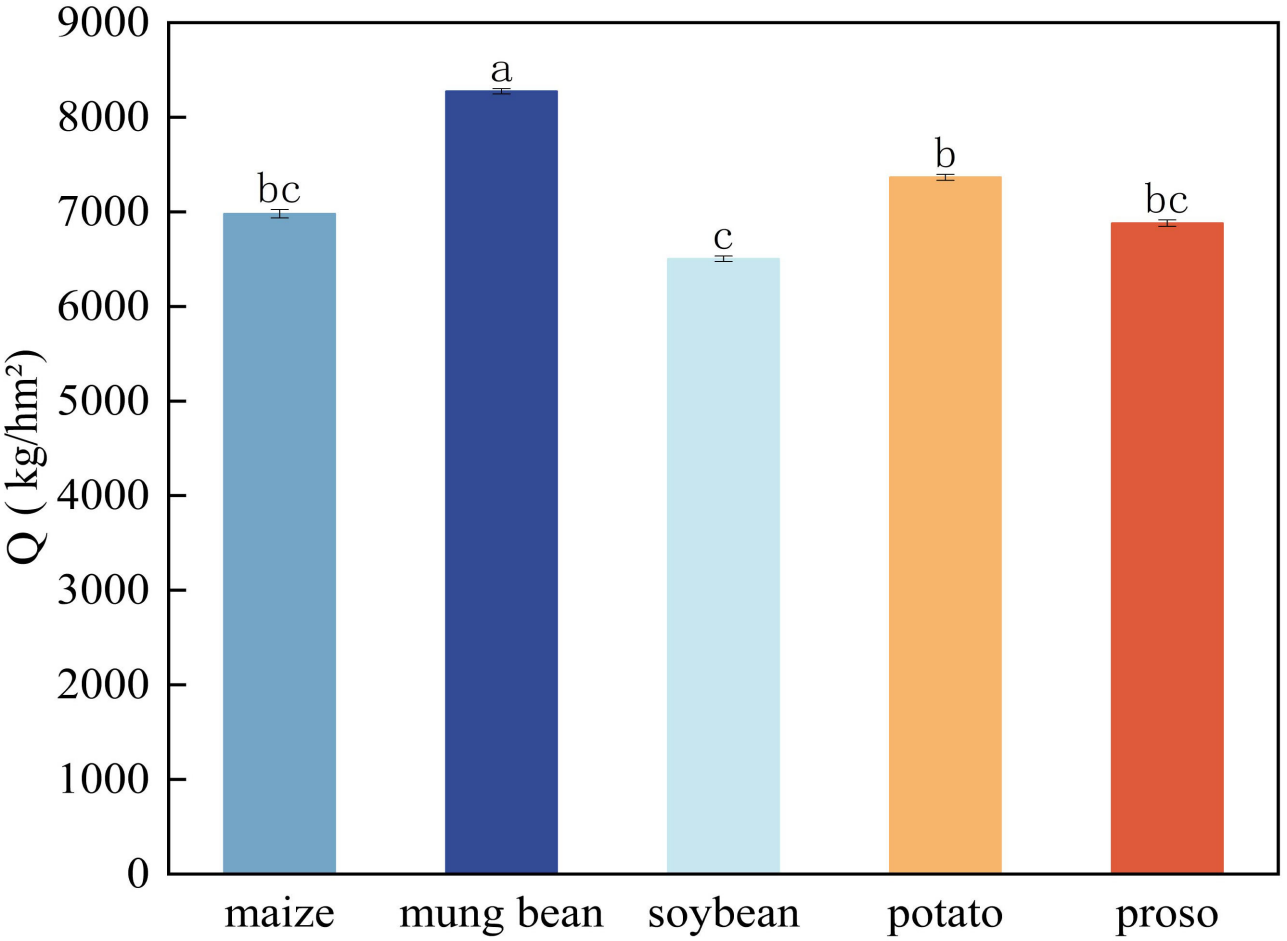
Impacts of different preceding crops on subsequent foxtail millet yield (Q). Values are mean ± SE of three replicates.Different letters (a, b, c, and d) represented significant differences (p < 0.05).

When the preceding crop was mung beans, the yield of foxtail millet (Q) reached its highest value of 8274.86 kg/hm^2^ (**Figure 2**), which significantly surpassed the yields obtained after the other treatments (*p*< 0.05). The second-highest yield of 7365.42 kg/hm^2^ was obtained when the preceding crop was potatoes, while the lowest yield of 6503.49 kg/hm^2^ was obtained when the preceding crop was soybean.

### 3.2 Impacts of different preceding crops on the quality of foxtail millet appearance

**Figure 3:**
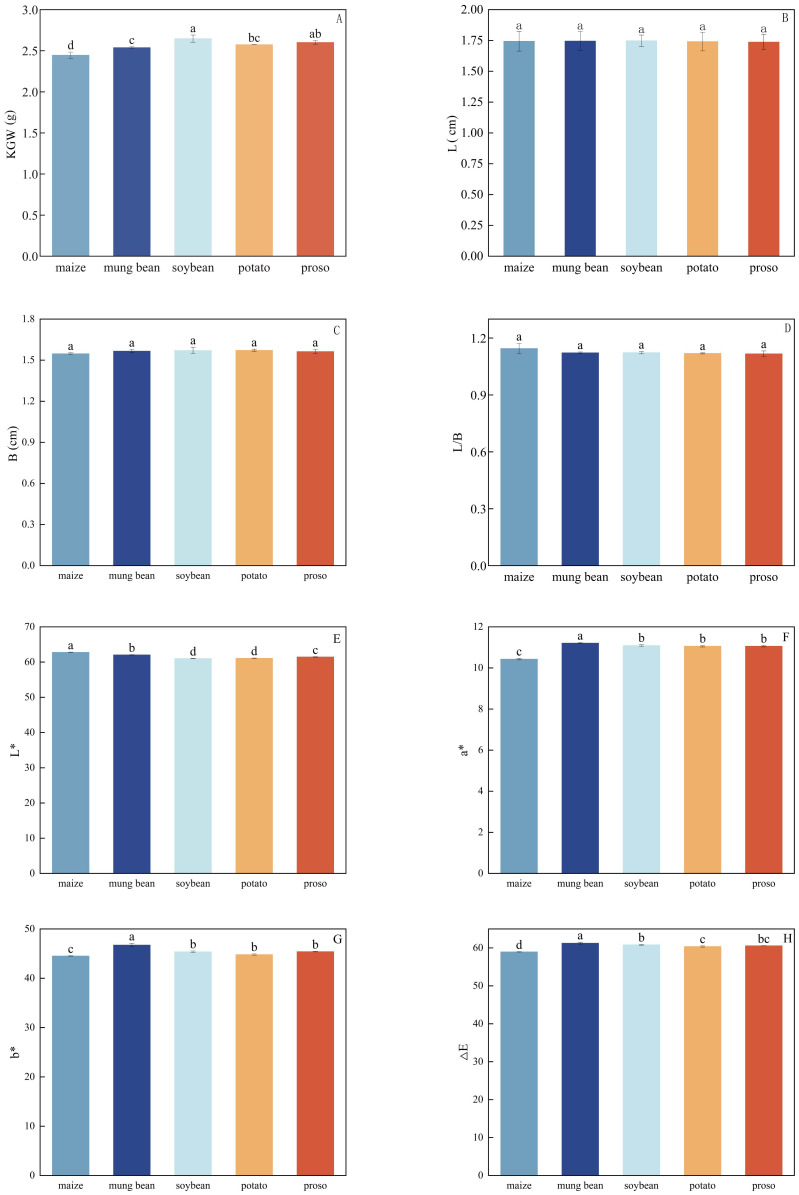
Impacts of different preceding crops on **(A)** 1000-grain-weight (KGW), **(B)** length (L), **(C)** breadth **(B)**, **(D)** L/B ratio, **(E)** brightness (L*), **(F)** red–green value (a*), **(G)** yellow–blue value (b*), and **(H)** the colour difference in foxtail millet (△E).Values are mean ± SE of three replicates. Different letters (a, b, c, and d) represented significant differences (p < 0.05).

The L, B, or L/B of the foxtail millet did not significantly differ under different preceding crops (**Figure 3**); however, KGW did present significant differences, with the highest value (2.648 g) observed when soybean was used as the preceding crop and the lowest (2.446 g) observed when maize was used. The KGW values were ordered soybean > proso millet > potato > mung bean > maize.

The colour characteristics of Zhangzagu10 were influenced by the preceding crop, with significant differences in L*, a*, b*, and △E for foxtail millet under different treatments. When the preceding crop was mung beans, the b* of foxtail millet was significantly higher than that of the other treatments, while the lowest b* was observed when the preceding crop was maize. However, when the preceding crop was maize, L* was the highest and significantly higher than that in other treatments, while it was the lowest when the preceding crop was soybeans.

### 3.3 Impacts of different preceding crops on the cooking quality of foxtail millet

**Figure 4:**
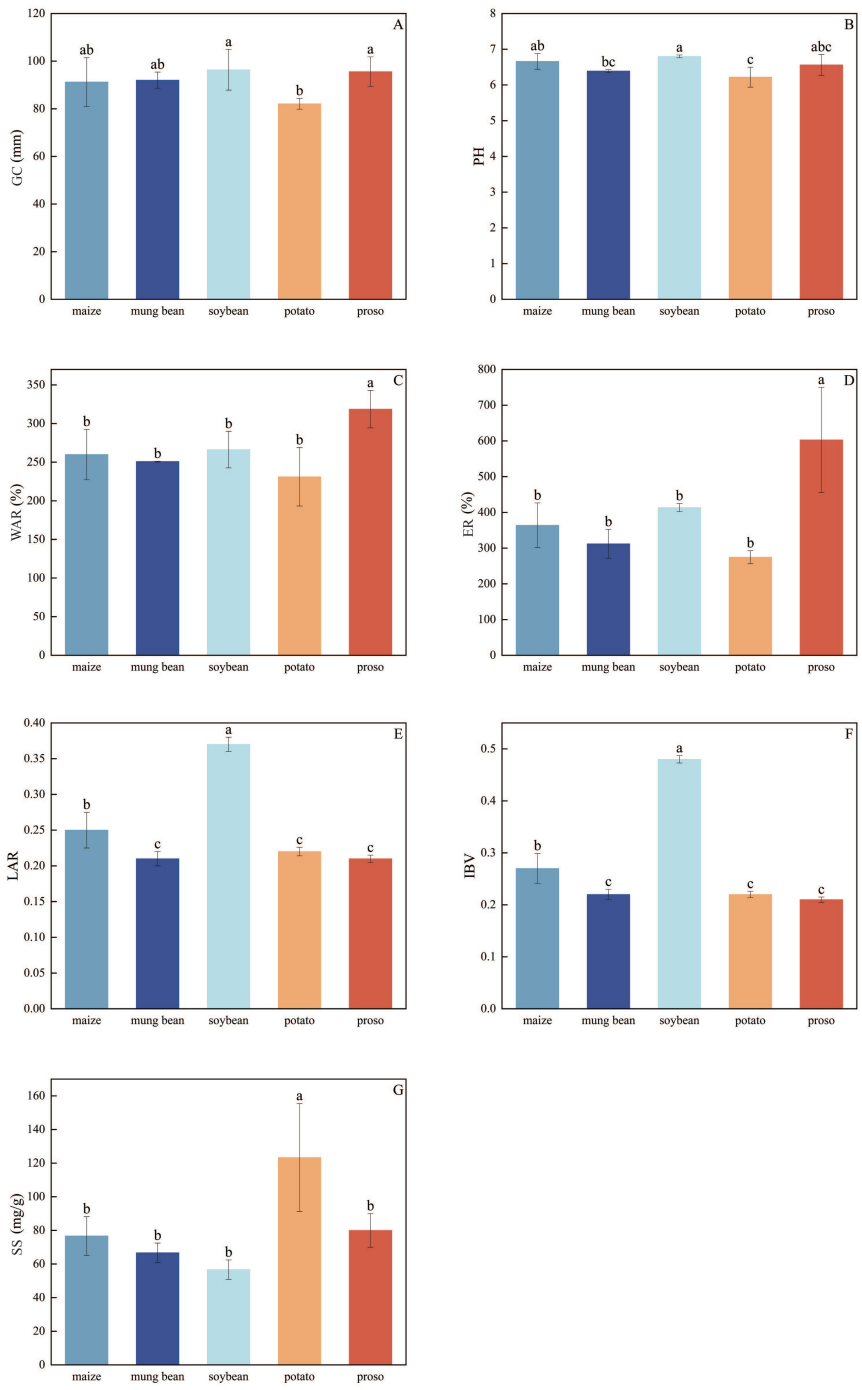
Impacts of different preceding crops on **(A)** gel consistency(GC), **(B)** pH, **(C)** water absorption rate (WAR), **(D)** expansion rate (ER), **(E)** absorbance of millet soup (LAV), **(F)** iodine-blue value (IBV), and **(G)** solid content of foxtail millet soup (SS).Values are mean ± SE of three replicates.Different letters (a, b, c, and d) represented significant differences (p < 0.05).

Zhangzagu10 had the highest gel consistency (GC) and pH, which were 93.36 mm and 6.80, respectively, when the preceding crop was soybeans (**Figure 4**). Meanwhile, when the preceding crop was potatoes, foxtail millet had the lowest GC and pH—82.10 mm and 6.22, respectively—which were significantly lower than those observed under soybean treatment. When the preceding crop was proso millet, Zhangzagu10 had the highest WAR and ER values, reaching 318.67% and 602.78%, respectively, which were significantly higher than those in the other four treatments. However, when the preceding crop was potatoes, Zhangzagu10 exhibited the lowest WAR and ER values (231.05% and 274.76%, respectively). Additionally, when the preceding crop was soybeans, Zhangzagu10 had the highest LAV and IBV, at 0.37 and 0.48, respectively, which were significantly higher than in the other treatments. Further, with the preceding proso millet, the foxtail millet had the lowest IBV (0.21).

The paste properties of Zhangzagu10 are listed in **Table 1**. There were no significant differences in the PV and PTM of foxtail millet under the different crop treatments. When the preceding crop was potatoes, the foxtail millet had the highest PV, FV, and SB and the longest PT, at 1110.67 cP, 2187.00 cP, 1076.33 cP, and 6.09 min, respectively. However, when the preceding crop was proso millet, Zhangzagu10 had the lowest PV, FV, and SB, at 835.67 cP, 1644.33 cP, and 808.67 cP, respectively. These values were significantly lower than those in the potato treatment. Mung beans, as the preceding crop, resulted in the highest BD value (259.33 cP) for foxtail millet, as well as the shortest gelatinisation time (5.76 min), which were significantly lower than those under potato rotation. When the previous crop was maize, Zhangzagu10 had the lowest BD (56.75 cP), which was significantly lower than that in the mung bean treatment.

**Table 1:** Impacts of different preceding crops on the pasting properties of foxtail millet.

	PV (cP)	TV (cP)	BD (cP)	FV (cP)	PT (min)	PTM (°C)	SB (cP)
Maize	988.25 ± 81.65	931.5 ± 67.17ab	56.75 ± 23.64b	1852.25 ± 117.75ab	6.03 ± 0.13a	87.21 ± 0.63	920.75 ± 50.89ab
Mung bean	1200.33 ± 394.48	941.00 ± 218.11ab	259.33 ± 179.53a	1850.67 ± 422.20ab	5.76 ± 0.15b	85.63 ± 2.14	909.67 ± 204.14ab
Soybean	1010.67 ± 70.29	908.00 ± 55.87ab	102.67 ± 24.01ab	1787.33 ± 96.11ab	6.00 ± 0.07a	88.02 ± 0.78	879.33 ± 42.00ab
Potato	1290.67 ± 361.82	1110.67 ± 227.20a	180.00 ± 135.64ab	2187.00 ± 434.79a	6.09 ± 0.19a	86.67 ± 2.35	1076.33 ± 207.73a
Proso millet	1008.33 ± 63.00	835.67 ± 47.06b	172.67 ± 16.26ab	1644.33 ± 88.92b	5.87 ± 0.07ab	86.43 ± 0.83	808.67 ± 41.86b
Max.	1708.00	1373.00	466.00	2689.00	6.20	88.80	1316.00
Min.	893.00	779.00	30.00	1543.00	5.67	83.20	764.00
SD	237.82	151.80	111.41	292.91	0.17	1.50	141.48

BD, breakdown viscosity; FV, final viscosity; PT, peak time; PTM, pasting temperature; PV, peak viscosity; SB, setback viscosity; SD, standard deviation; TV, total viscosity. Different letters (a, b, c, and d) indicate significant differences (*p<* 0.05).

### 3.4 Impacts of different preceding crops on the nutritional quality of foxtail millet

**Figure 5:**
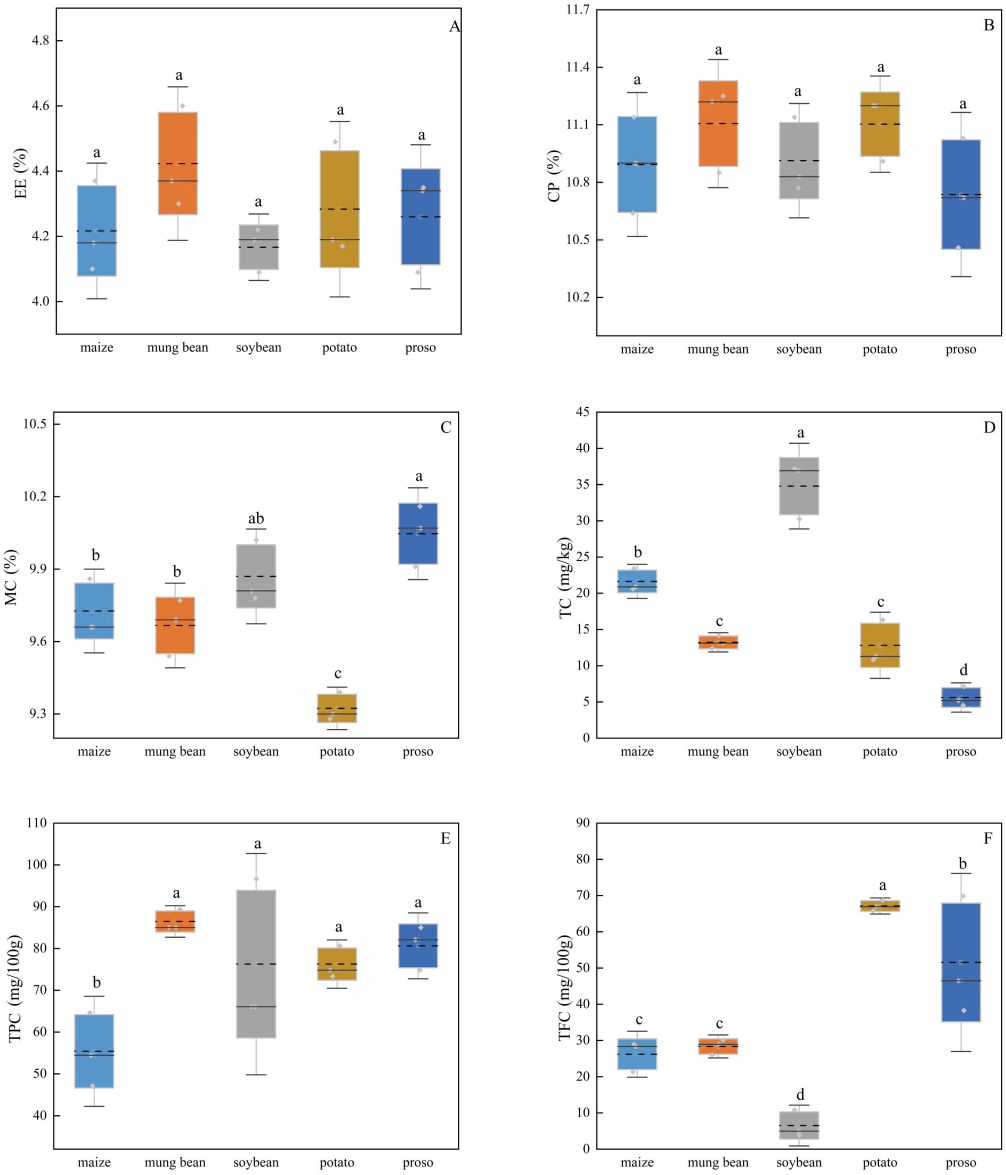
Impacts of different preceding crops on **(A)** crude fat (EE), **(B)** crude protein (CP), **(C)** moisture content (MC), **(D)** total carotenoids (TC), **(E)** total polyphenols (TPC), and **(F)** total flavonoids (TFC).The values reported are mean ± standard error of three replicates.Different letters (a, b, c, and d) represented significant differences (p < 0.05).

As shown in **Figure 5**, different preceding crops influenced various nutrient components of the foxtail millet. No significant differences were observed in the crude fat (EE) or crude protein (CP) contents of the foxtail millet among the different treatments. When the preceding crop was proso millet, Zhangzagu10 had the highest moisture content (MC = 10.05%), and when the preceding crop was potato, it had the lowest MC (9.32%), which was significantly lower than that of the other four crops.

Zhangzagu10 had the highest TC (34.79 mg/kg) when the preceding crop was soybeans, and this was significantly higher than the TC values under other crop treatments. When the preceding crop was proso millet, Zhangzagu10 had the lowest TC, at only 5.61 mg/kg, which was significantly lower than the values under the other treatments. Meanwhile, mung beans yielded the highest foxtail millet TPC of 86.46 mg/100 g, which was significantly higher than that under the other crop treatments. When the preceding crop was maize, foxtail millet had the lowest TPC (55.41 mg/100 g), which was significantly lower than that under the other treatments.

**Figure 6:**
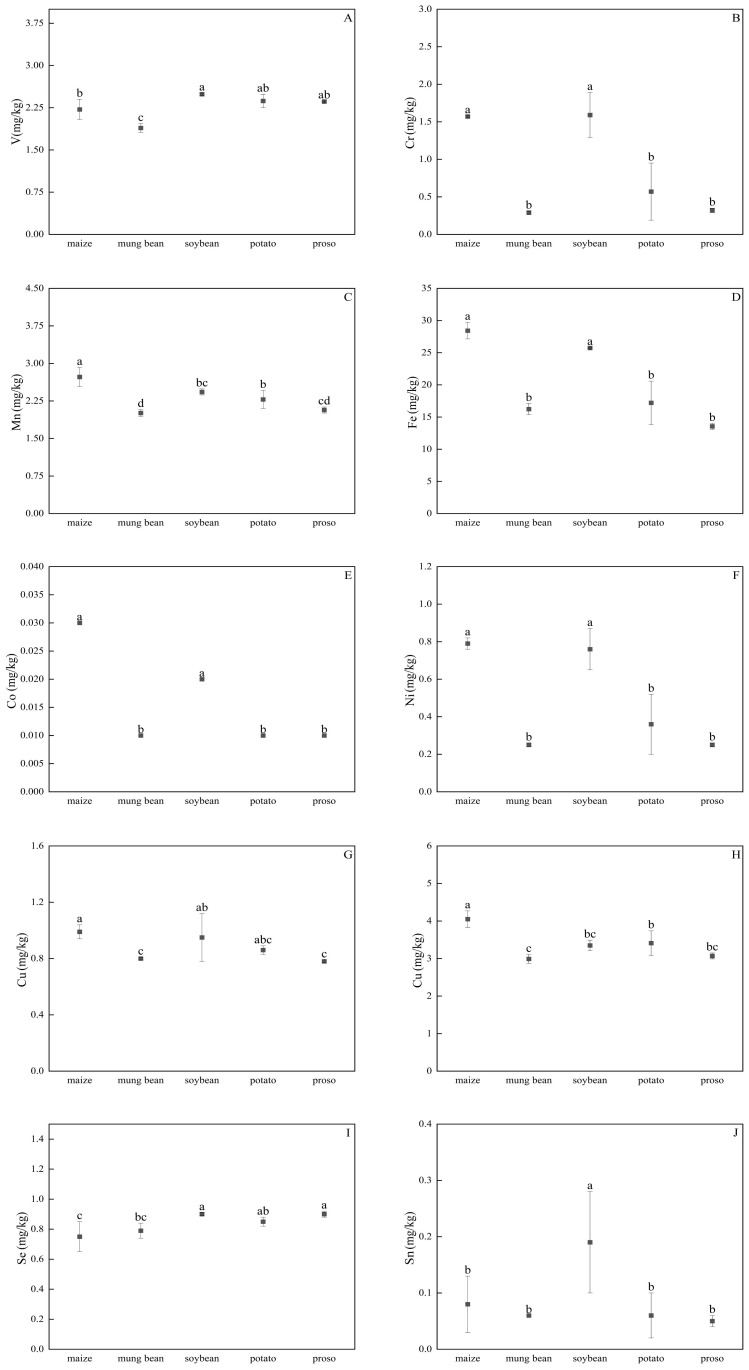
Impacts of different preceding crops on the following elements: **(A)** V, **(B)** Cr, **(C)** Mn, **(D)** Fe, **(E)** Co, **(F)** Ni, **(G)** Cu, **(H)** Zn, **(I)** Se, **(J)** Sn. Values are mean ± SE of three replicates. Different letters (a, b, c, and d) represented significant differences (p < 0.05).

Preceding potatoes resulted in the highest total flavonoid content (TFC; 67.13 mg/100 g) in the foxtail millet, which was significantly higher than that under the other four treatments. When the preceding crop was soybeans, the TFC was the lowest, at only 6.53 mg/100 g, which was significantly lower than that under the other treatments. With the preceding soybean treatment, Zhangzagu10 had higher V, Cr, Fe, Co, Ni, Se, Sn, Mn, Cu, and Mo contents than those under the other treatments (**Figure 6**). Meanwhile, with the proso millet treatment, the contents of all measured elements except for Sn were lower than those in the other treatments.

### 3.5 Correlation between soil nutrients and foxtail millet yield and quality

**Figure 7:**
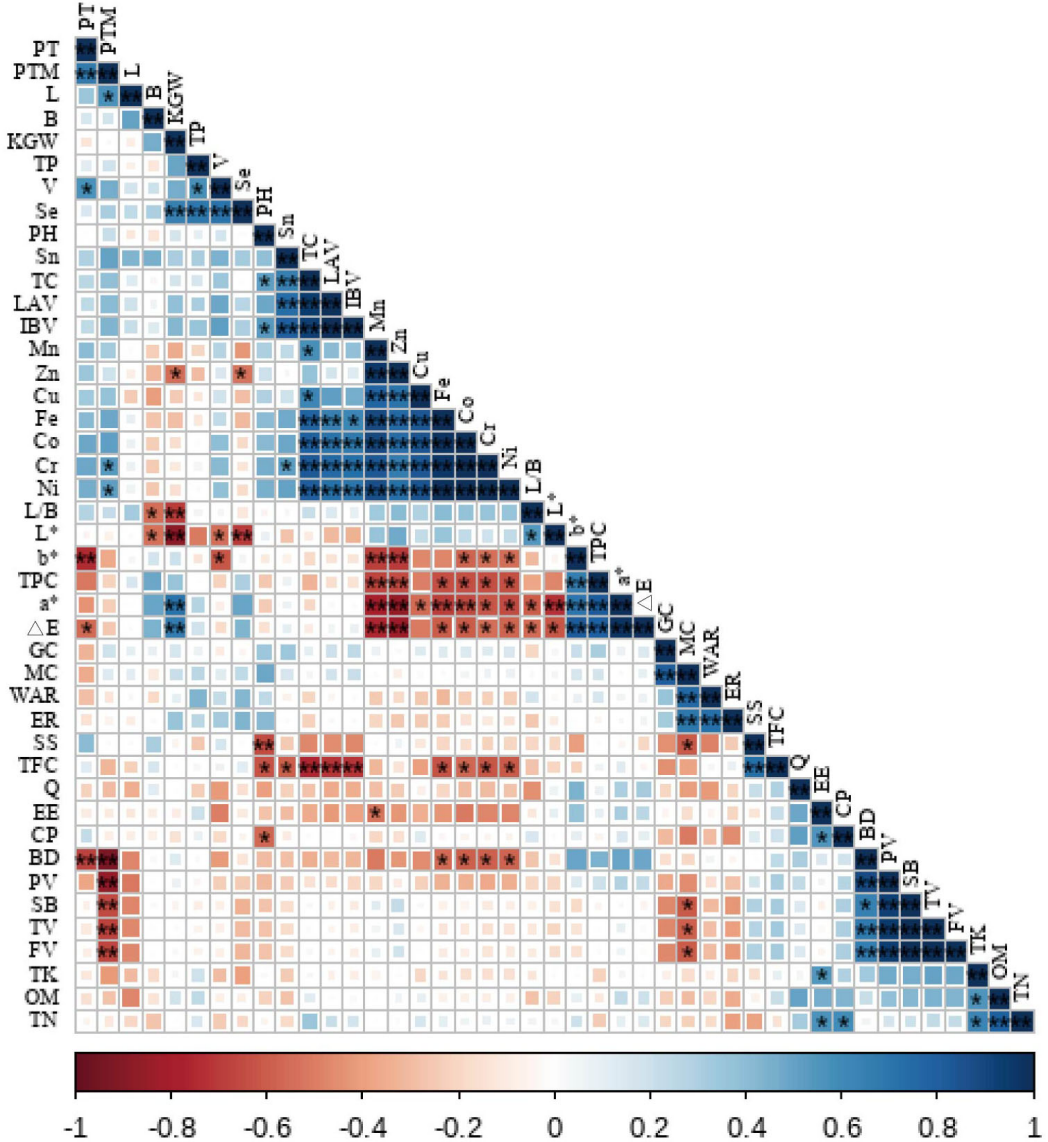
Correlation of foxtail millet yield and soil nutrient status. BD, breakdown viscosity; FV, final viscosity; SB, setback viscosity; TV, trough viscosity; PT, peak time; PTM, pasting temperature; PV, peak viscosity.

Correlation analysis revealed different relationships between foxtail millet yield and soil nutrient indicators (**Figure 7**). The following indicators were positively correlated with one another: OM–TN, OM–TK, TN–TK, TN–EE, TN–CP, TK–EE, TP–V, and TP–Se. KGW was significantly positively correlated with a*, △E, and Se, and significantly negatively correlated with L/B and L*. GC was directly proportional to MC, while the WAR and ER were both directly proportional to MC. TC was positively correlated with elements, though TFC and TPC were negatively correlated with elements, and there was a strong positive correlation among the elements.

### 3.6 Clustering of foxtail millet quality

**Figure 8:**
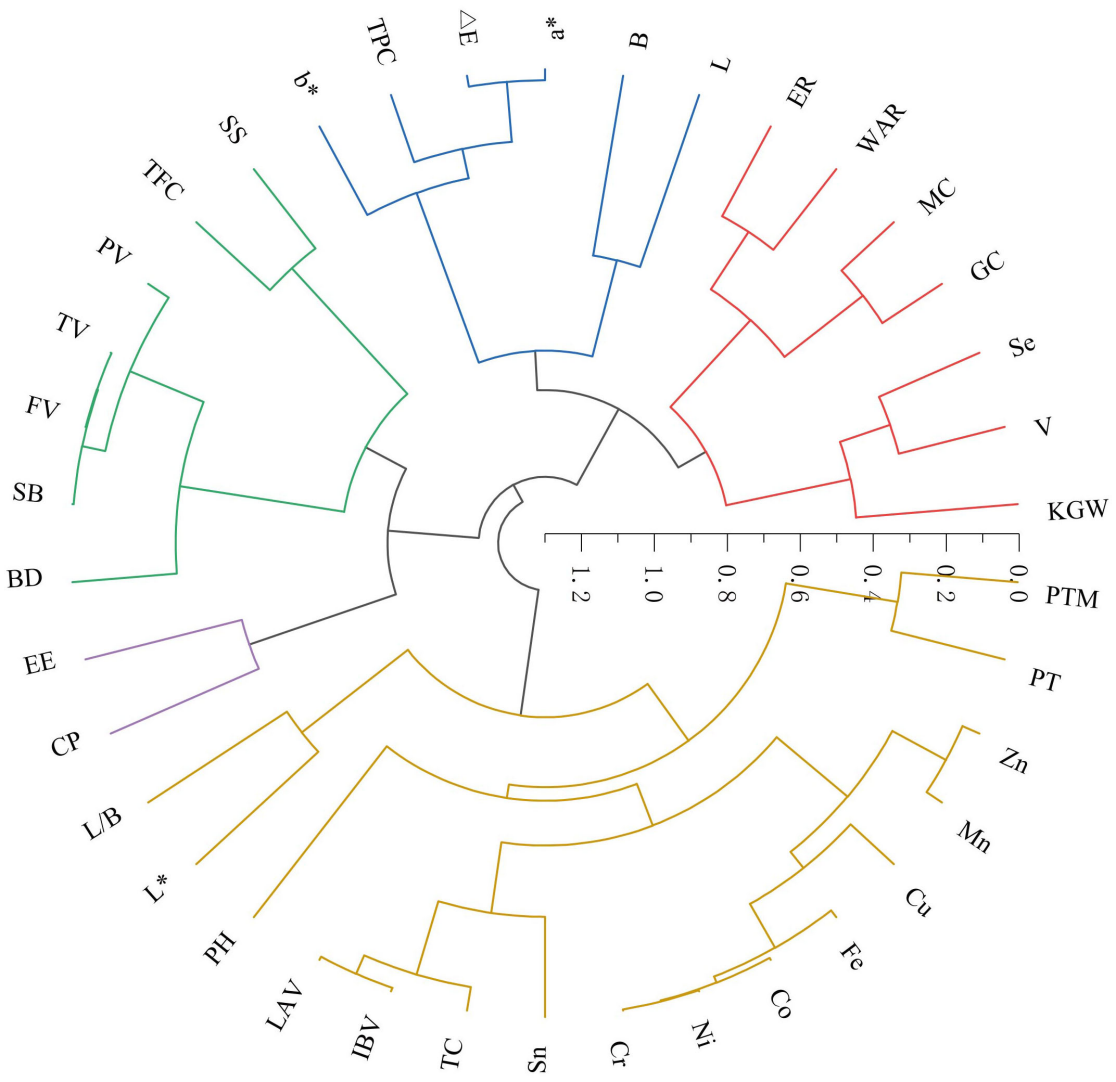
Hierarchical clustering of 38 commercial quality parameters.

The 38 foxtail millet quality parameters assessed in this study clustered into five groups (**Figure 8**). Group I consisted of L, B, a*, △E, b*, BD, and TPC. Group II included SS, TFC, PV, TV, FV, and SB. Group III comprised EE and CP. Group IV was composed of L/B, L*, pH, LAV, IBV, TC, Sn, Cr, Ni, Co, Fe, Cu, Mn, Zn, PT, and PTM. The remaining seven traits (KGW, V, Se, GC, MC, WAR, and ER) belonged to Group V.

## 4 Discussion

### 4.1 Soil nutrients under different preceding crops

Soil nutrient content plays a crucial role in maintaining soil fertility, supporting crop growth, and managing agricultural practices and represents one of the key indicators for assessing soil productivity (Guo et al., 2021). Appropriate crop rotation promotes the long-term stability of organic matter, which positively influences soil properties, crop productivity, and agricultural ecosystem sustainability (Skinulienė et al., 2024). This study aimed to investigate the effects of five preceding crops on soil nutrient content. Our findings indicate that when potatoes are used as the preceding crop, the soil exhibits the highest organic matter content, possibly due to the decomposition of potato root residues. In our research, the rotation of foxtail millet with leguminous crops (mung bean and soybean) resulted in higher total nitrogen and potassium levels in the soil, suggesting that the cultivation of legumes enhances the availability of these nutrients. This effect may be attributed to the nitrogen-fixing capabilities of legumes and the contribution of their roots to improving soil structure. Previous studies have shown that intercropping potatoes with soybeans can increase soil organic matter content by 12–28% compared to monoculture of potatoes (Nyawade et al., 2019). This increase is likely due to legumes enhancing soil respiration, which boosts the transformation of organic matter (Nyawade et al., 2019). Leguminous crops also contribute to increasing soil nitrogen reserves (Sharma et al., 1985). Furthermore, rotating with leguminous crops can enhance soil multifunctionality and help maintain organic carbon reserves (Liu et al., 2023).

However, the limited duration of this study hindered our ability to capture the long-term trends in soil nutrient changes. As the crop rotation cycle is extended, both organic carbon and total nitrogen levels consistently increase (Zhao et al., 2020). Long-term crop rotation not only improves the physicochemical properties of the soil but also enhances enzyme activity and microbial diversity (Guo et al., 2024). In the future, we will conduct long-term field experiments to assess the lasting effects of different preceding crops on soil nutrients and explore the interaction mechanisms between crops and soil microorganisms.

### 4.2 Grain yield of foxtail millet under different preceding crops

When mung beans were the preceding crop, millet presented the highest yield, which was likely due to the nitrogen-fixing properties of mung beans and their ability to improve the soil structure (Galieni et al., 2017). Previous reports indicate that mung beans can increase the grain yield of subsequent crops (Ilyas et al., 2018), such as wheat (Nitya et al., 2018) and that a mung bean-corn rotation can enhance the yield of dryland corn (Jaya et al., 2021). Leguminous crops can promote the growth of subsequent crops by fixing nitrogen and improving soil conditions (Arroyo et al., 2022), which is consistent with the high yield results observed in this study with mung beans. Notably, when soybeans were used as the preceding crop, the yield of millet was at its lowest. This finding contradicts previous research, which suggested that summer soybean rotations can enhance the yield of subsequent winter wheat (Jin et al., 2022). The reason for this discrepancy may be that soybeans deplete soil nutrients more significantly during their growth, thereby failing to effectively boost the yield of subsequent millet; however, this warrants further investigation.

In general, using the same crop as the preceding crop is the least suitable for subsequent crops due to the similar nutritional demands (Hlisnikovský et al., 2024). In this study, it was found that the yields of foxtail millet were relatively low when rotated with proso millet and maize. This may be attributed to the fact that these three crops belong to the Poaceae family and share similar growth habits. In the future, we will explore the effects of different crop root systems on soil microbial communities, nutrient dynamics, and water utilisation to investigate the specific mechanisms influencing the growth of subsequent millet.

### 4.3 Quality of foxtail millet under different preceding crops

The appearance of grain significantly influences consumers’ perceptions and purchasing decisions (Zhao et al., 2022). The thousand-kernel weight is a crucial metric for measuring the weight of grain kernels (Wu et al., 2018) and reflects the fullness and size of the grains. In this study, when soybeans were used as the preceding crop, Zhangzagu10 exhibited the highest thousand-kernel weight and had L and B values that were relatively long, indicating that the grains were round and plump. This aligns with previous research, which found that leguminous crops can enhance the thousand-kernel weight of subsequent crops (Ingver et al., 2018). The parameters L*, a*, and b* represent brightness, red-green hue, and yellow-blue hue, respectively. When mung beans were the preceding crop, the b* value was highest, indicating that the grains appeared yellow. These results suggest that the choice of preceding crops affects the physical characteristics of the foxtail millet and may also influence the sensory quality.

Cooking quality and paste characteristics play a crucial role in the marketability of foxtail millet (Chowdaiah et al., 2022). High-quality millet is characterised by a soft, sticky texture, the absence of hard kernels, and a pleasant aroma (Nitya et al., 2018). When foxtail millet exhibits higher viscosity, the resulting soup or porridge becomes thicker. Foxtail millet with a higher water absorption ratio (WAR) and expansion ratio (ER) tends to have a softer and richer texture after cooking (Faruq et al., 2015). Amylose content serves as a source of resistant starch, which can help regulate blood sugar levels and improve gut health (Li et al., 2023). Foxtail millet with low amylose content (< 20%) is generally softer and stickier when cooked (Arroyo et al., 2022). In addition, the integrity of the cooked millet is directly proportional to its amylose content (Ma et al., 2023). Higher peak time (PTM) values can extend cooking times, waste energy, and potentially lead to inferior texture, whereas lower PTM values are associated with better cooking quality (Li et al., 2023). The swelling power (SB) is positively correlated with grain hardness and negatively correlated with viscosity. When the SB value is negative, the millet becomes overly sticky, while higher positive values indicate harder, coarser grains. Under conditions of crop rotation with mung beans, Foxtail millet exhibits the lowest integrity before cooking (IBV), along with higher WAR and ER, the highest bulk density (BD), and a positive but relatively low SB value, resulting in a thick and soft porridge. Generally, higher peak viscosity (PV) reflects the expansion capability of starch granules, and varieties of foxtail millet with better texture tend to have higher PV and total viscosity (TV) values (Ma et al., 2023). When potatoes are used as the preceding crop, foxtail millet shows the highest PV and TV, resulting in a soft texture. In contrast, when maize is the preceding crop, foxtail millet has the lowest BD and a larger PTM, leading to a hard texture and poor palatability.

Foxtail millet is an important crop in China that is rich in various nutrients (Wang et al., 2023), and it holds particular significance for the people in northern China. In addition to factors such as genotype and environment, crop rotation has been shown to influence the nutritional quality of plants (Xia et al., 2023). The carotenoids in foxtail millet play a crucial role not only in determining the colour of the grains but also in providing antioxidant, anti-ageing, visual protection, and immune-enhancing effects (Torsten et al., 2023). When soybeans were used as the preceding crop, the carotenoid content in Zhangzang10 was highest. Flavonoids can scavenge free radicals in the body, improve blood circulation, and offer various health benefits, including reducing the burden of cardiovascular diseases (Socci et al., 2017). When potatoes are used as the preceding crop, the flavonoid content is highest. Polyphenols exhibit strong antioxidant properties by reducing oxidative damage and playing a vital role in protecting cells and tissues (Giovinazzo and Grieco, 2015). When mung beans were the preceding crop, the polyphenol content was highest. Soybeans, mung beans, and potatoes all contributed positively to the nutritional quality of foxtail millet, with legumes being particularly beneficial.

Many elements are essential components of the human body, and based on their effects on human health, they can be categorised into beneficial and toxic groups. Elements such as Ca, K, Fe, Cu, Zn, Mn, Se, Mo, Ni, Cr, V, Co, and Sn are beneficial to humans, with selenium being particularly important. Notably, foxtail millet and its products serve as the primary source of selenium intake for humans (Xie et al., 2021). In this study, when soybeans were used as the preceding crop, the contents of Cr, V, Co, Se, and Sn in foxtail millet were highest. The findings of this study support existing research on the impact of crop rotation on the mineral element content. Previous literature has indicated that different preceding crops can significantly enhance the mineral composition of subsequent crops by improving soil properties and nutrient availability (Fernández et al., 2020; Song et al., 2021). Leguminous crops in particular can increase the bioavailability of nitrogen in the soil due to their nitrogen-fixing capabilities (Peoples et al., 2009). Furthermore, studies focusing on the accumulation of mineral elements in cereal crops have also demonstrated the influence of preceding crops on the nutritional composition of millet (Silke et al., 2018). Therefore, the findings of this study further validate these prior research results, underscoring the critical role of preceding crops in enhancing the mineral nutrition of subsequent crops. This study specifically investigated the accumulation of beneficial elements in mature foxtail millet under different crop rotation patterns. Previous research has shown that compared to rotations with wheat, rotations with leguminous crops can enhance the bioavailability and absorption of cadmium (Cd) (Tillmann et al., 2016). Thus, further research should explore the absorption and distribution of these elements as well as the potential uptake of toxic elements.

## 5 Limitations

This research, however, is subject to several limitations. First, the selection of experimental fields in this experiment is relatively simple, covering only the specific soil types and climate conditions in the area, which may limit the general applicability of the research results; field experiments are susceptible to the natural environment (such as climate change, natural disasters) and market factors and other external factors, which will affect the research results. Second, due to time and resource constraints, we were not able to study the long-term effects of crop rotation years on soil nutrition and foxtail millet quality. Therefore, in the future, it is recommended to conduct similar studies in a wider range of geographical and climatic conditions and extend the study period to fully assess the long-term effects of different previous crops on soil and foxtail millet quality.

## 6 Conclusions

The effects of different preceding crops on foxtail millet varied. Foxtail millet productivity was the highest when mung beans were used as the preceding crop; moreover, the foxtail millet grains exhibited the most yellow colouration and cooking quality. When soybeans were used as a preceding crop, foxtail millet grains are plump and nutritious, whereas when potatoes were used as the preceding crop, poor gelatinisation but high nutrient density were observed. When either maize or proso millet was used as the preceding crop, all the measured qualities were relatively poor. Our findings suggest that leguminous crops (mung beans and soybeans) are more suitable as preceding crops for foxtail millet cultivation, whereas gramineous crops (maize and proso millet) are not suitable for inclusion in foxtail millet rotation systems.Based on this, we hypothesize that:1) The beneficial effects of leguminous crops on millet could be due to enhanced soil nitrogen levels and improved soil health.2) The poorer quality associated with maize and proso millet may stem from lower nutrient availability or competition effects.Further research is recommended to explore the mechanisms behind these effects, such as soil nutrient dynamics and plant interactions. Additionally, field trials with different combinations of preceding crops could provide deeper insights into optimizing foxtail millet production systems.

## Data Availability

The raw data supporting the conclusions of this article will be made available by the authors, without undue reservation.
